# Multiplex biomarker-based ELISA enables early detection of *Mycobacterium avium* subspecies *paratuberculosis*-specific antibodies comparable to IFN-*γ* testing

**DOI:** 10.3389/fvets.2025.1749510

**Published:** 2026-01-20

**Authors:** Shih-Jiuan Chiu, Piyush Bugde, Andrea Kinga, John P. Bannantine, Simon Liggett, Venkata Sayoji Rao Dukkipati, Yoichi Furuya

**Affiliations:** 1 Pictor Limited, Auckland, New Zealand; 2 US Department of Agriculture-Agricultural Research Service, National Animal Disease Center, Ames, IA, United States; 3 Disease Research Limited, Invermay Agricultural Centre, Mosgiel, New Zealand; 4 Shreiber School of Veterinary Medicine, Rowan University, Glassboro, NJ, United States; 5 School of Agriculture and Environment, Massey University, Palmerston North, New Zealand

**Keywords:** antibodies, biomarker, cattle, Johne’s disease, MAP, multiplex ELISA, *Mycobacterium avium* subspecies paratuberculosis

## Abstract

Johne’s disease, caused by *Mycobacterium avium* subspecies *paratuberculosis* (MAP), continues to pose a major global challenge for the livestock industry due to its long subclinical phase and the limitations of current diagnostics. Commercial antibody-based ELISA tests often fail to detect early-stage infections, while interferon (IFN)-*γ* assays, though considered more sensitive at early stages, are costly and logistically complex. We present a novel multiplex ELISA that incorporates multiple MAP antigens for the detection of MAP-specific IgG antibodies in bovine serum and milk. Using well-characterized positive and negative cohorts, the assay demonstrated strong concordance with IFN-*γ* responses and outperformed commercial monoplex ELISA kits (IDEXX and IDVET). Our findings support its potential as a practical, high-throughput alternative for early detection of MAP-specific antibodies and for herd-level disease management.

## 1 Introduction

Johne’s disease is a chronic, progressive gastrointestinal infection in ruminants caused by the intracellular pathogen *Mycobacterium avium* subspecies *paratuberculosis* (MAP). The disease is prevalent worldwide, with higher incidence rates observed in regions practicing intensive livestock farming (1). Although infection typically occurs in young animals through ingestion of MAP-contaminated milk, water, or feed, clinical signs often do not manifest until later in life (2). This prolonged asymptomatic or subclinical phase enables infected animals to silently shed MAP into the environment, contributing to within- and between-herd transmission (3), with economic losses estimated to exceed US$250 million annually due to reduced milk production, premature culling, and impaired reproductive performance (4, 5).

Early detection of MAP-infected animals is essential for controlling Johne’s disease, enabling timely intervention, limiting transmission, and mitigating economic losses. Effective control strategies depend on accurate screening tools capable of identifying infections before they reach advanced clinical stages or become high shedders. Current diagnostics for Johne’s disease fall into two broad categories: direct detection of MAP (e.g., culture or qPCR) and indirect detection through host immune responses (e.g., ELISA) (6). While direct methods are highly specific, they are also labour-intensive, costly, and unsuitable for high-throughput herd screening. ELISAs are widely used due to their cost-effectiveness and practicality, particularly when applied to milk or serum samples. Milk ELISAs, in particular, are favoured in field settings due to the ease and lower cost of sampling (7). However, the sensitivity of conventional monoplex ELISA tests, such as those offered by IDEXX’s MAP Ab Test Paratuberculosis (Johne’s Disease)/*Mycobacterium avium* subsp. *paratuberculosis* (MAP) and IDVET’s ID Screen® Paratuberculosis Indirect, hereon referred to as IDEXX and IDVET ELISA, is limited, especially in early stage infections when antibody levels are low (8, 9). These limitations often lead to false-negative results, which enable infected animals to remain undetected and continue spreading the disease. Consequently, there is an urgent need for improved diagnostic assays with greater sensitivity, reliability, and suitability for early detection (10).

In this study, we present the development of the Pictor PictVet™ *Mycobacterium avium* subspecies *paratuberculosis* IgG Multiplex ELISA, a novel immunoassay designed to improve diagnostic sensitivity, particularly in subclinical cases, by incorporating multiple MAP-specific antigens and broadening the antibody detection profile. To evaluate its performance, both serum and milk samples from well-characterized positive and negative cohorts were analyzed. The assay’s results were compared with those from commercial test kits (IDEXX and IDVET ELISA), using the interferon (IFN)-*γ* assay as a comparator test. Our findings support the assay’s potential as a high-throughput, practical tool for early detection of MAP antibodies and effective management of Johne’s disease in dairy herds.

## 2 Materials and methods

### 2.1 Bovine milk and serum samples

The development of the Pictor PictVet™ MAP IgG Multiplex ELISA was based on a set of 15 heat-treated paired serum and milk samples. These samples were characterized using two commercially available MAP Ab tests (IDEXX and IDVET) and an in-house interferon-gamma (IFN-*γ*) assay (11). The IDVET ELISA served as the comparator test, while the IFN-γ test served as the reference method. The IDVET ELISA was performed according to the manufacturer’s Instructions for Use. For this study, 12 paired serum and milk samples were selected from previously collected samples obtained from cattle naturally exposed to MAP. Three negative control samples (serum and milk) were obtained from healthy animals with no history of disease. The samples were obtained from female Holstein cattle ranging in age from 5 to 11 years. All samples were stored at −80 °C until analysis. The date of collection for each paired milk and serum sample, along with their animal IDs and health status, is provided in Table 1.

**Table 1:** Collection dates of serum and milk samples used in this study.

Sample ID	Milk collection date	Serum collection date	Health status^a^
785	03/01/2011	20/09/2011	Control
486	20/04/2022	13/04/2022	Control
488	20/04/2022	13/04/2022	Control
781	03/01/2011	29/03/2011	Suspect
1,091	19/11/2012	16/10/2012	Suspect
9,514	14/11/2012	16/10/2012	Subclinical
788	27/10/2013	2/05/2013	Subclinical
1,044	14/11/2012	16/10/2012	Subclinical
1,459	29/112010	20/05/2010	Subclinical
1,618	20/04/2022	13/04/2022	Subclinical
1701	20/04/2022	13/04/2022	Subclinical
6,607	20/04/2022	20/04/2022	Subclinical
2,222	25/04/2012	16/10/2012	Clinical
6,739	20/04/2022	13/04/2022	Clinical
6,878	20/04/2022	20/04/2022	Clinical

^a^Health status classifications were determined using fecal culture, PCR, antibody ELISA, and IFN-γ assays. Animals labeled as “suspect” exhibited intermittent IFN-γ positivity with consistently negative antibody results, which is often indicative of very early subclinical disease. Specifically, animal 781 showed IFN-γ positivity on 19/11/2009 and 13/03/2012 but was negative on seven other sampling dates between 27/03/2010 and 15/11/2013. Animal 1,091 was intermittently IFN-γ positive on 20/11/2011, 16/10/2012, 02/04/2013, and 28/04/2014, but negative on five other occasions. All samples were sourced from the USDA facility. Some animals were naturally exposed to MAP, others are healthy controls and samples were collected at the indicated time points. All samples were sourced from the USDA facility.

### 2.2 MAP antigens

Recombinant MAP2609c, MAP1569c, and MAP1138c proteins were custom generated by GenScript (Piscataway, NJ). All three antigens were expressed in *E. coli* and purified from the supernatant of cell lysates using a GST affinity column, followed by removal of the GST or MBT fusion tags according to the manufacturer’s specifications. MAP2609c, MAP1569c, and MAP1138c were supplied at purities of ≥60 percent, ≥85 percent, and ≥90 percent respectively, based on SDS PAGE under reducing conditions. Each antigen was provided in a defined storage buffer (50 mM Tris HCl, 150 mM NaCl, 10 percent glycerol, pH 8.0) and stored at −80 °C. Antigen identity and functional activity were further verified on the Pictor PictVet™ MAP IgG Multiplex ELISA using MAP positive bovine reagents (Allied Monitor, Fayette, MO). In addition to the recombinant proteins, Paratuberculosis protoplasmic antigen (PPA; Allied Monitor, Fayette, MO) was included as a lyophilised protoplasmic extract of MAP, prepared according to the supplier’s recommended rehydration and ELISA working dilutions. Johnin (Disease Research Laboratory, New Zealand), a heat killed purified protein derivative derived from MAP, was also included in the antigen panel. Together, these antigens were used as coating antigens on the Pictor multiplex ELISA platform.

### 2.3 Pictor PictVet™ MAP IgG multiplex ELISA assay

**Figure 1:**
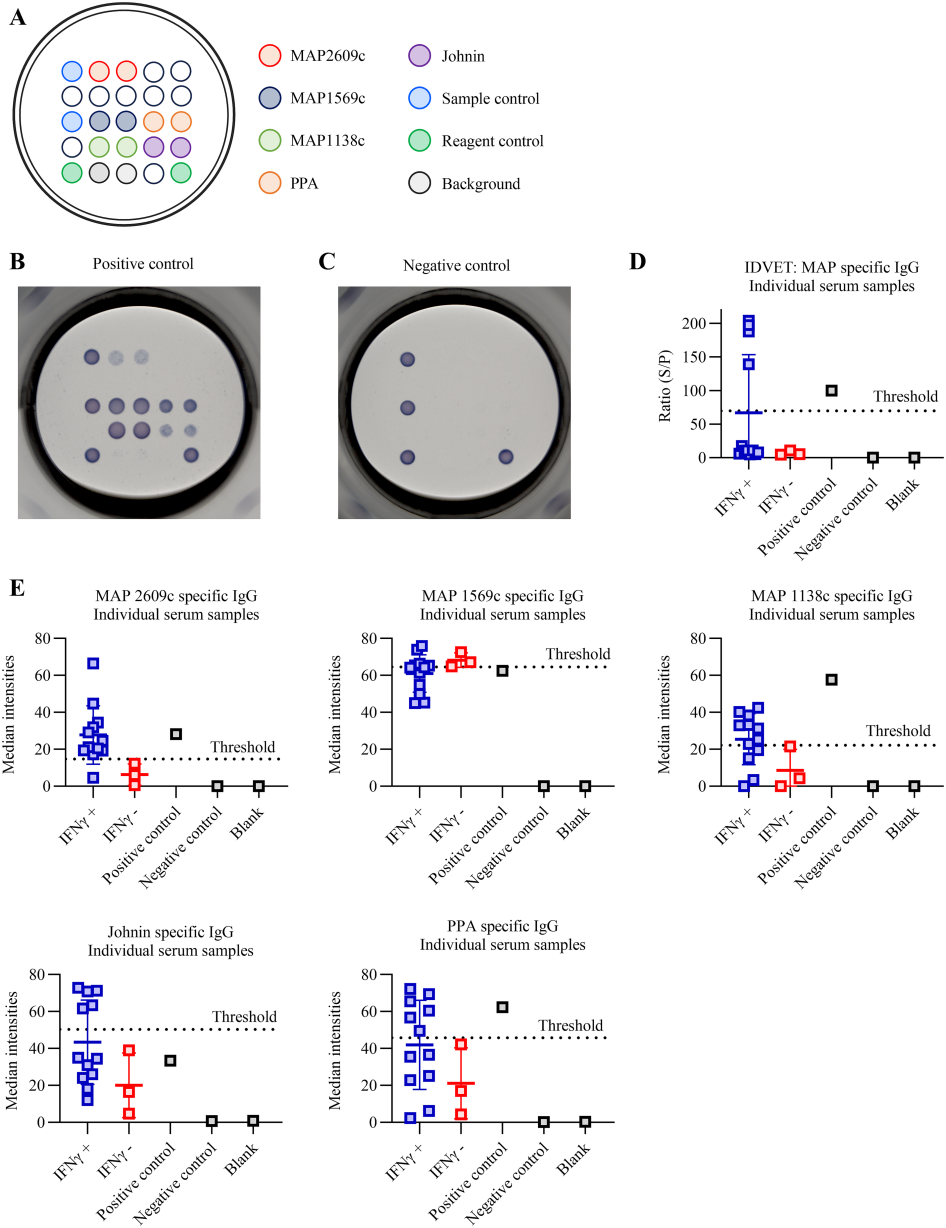
Pictor PictVet™ MAP IgG multiplex ELISA detects MAP-specific IgG antibodies in bovine serum. **(A)** Layout of the Pictor PictVet™ MAP IgG multiplex ELISA plate showing the printed antigens MAP2609c, MAP1569c, MAP1138c, Johnin, and PPA. Internal controls for sample validity (sample control), reagent performance (reagent control), and print buffer (background) were also included. **(B,C)** Representative assay images obtained using positive **(B)** and negative **(C)** control reagents (bovine lyophilized sera; Allied Monitor, Fayette, MO). **(D)** IDVET ELISA results for serum samples categorized as IFN-*γ*–positive and IFN-γ–negative. **(E)** Scatter plots showing the distribution of signal intensities for antibodies specific to MAP2609c, MAP1569c, MAP1138c, Johnin, and PPA in IFN-*γ*–positive (*n* = 11; blue data points) and IFN-*γ*–negative (*n* = 4; red data points) bovine serum samples. The dotted lines indicate the assay cut-off values for each antigen. Error bars represent standard deviation (SD).

PictArray™ Multiplex ELISA plates were prepared by spotting 10 nL of recombinant MAP antigens: MAP2609c, MAP1569c, MAP1138c, PPA, and Johnin onto 96-well ELISA plates, as illustrated in Figure 1A. Anti-bovine IgG and anti-HRP IgG were co-printed as internal positive controls, while print buffer spots served as controls to monitor background signal arising from buffer components or potential antigen carryover. All antigen spots were printed in duplicate. Following printing, plates were incubated with a blocking solution to minimize non-specific antibody binding. The assay was performed using a standard indirect ELISA protocol, following the instructions provided with the Pictor PictVet™ MAP IgG Multiplex ELISA kit. Briefly, serum and milk samples were diluted 1:100 and 1:20, respectively, and 100 μL of each diluted sample was added to individual wells, followed by incubation at 37 °C for 30 min. Plates were washed three times before the addition of 100 μL biotinylated detection antibody for 30 min at 37 °C. After an additional three washes, 100 μL of streptavidin-HRP was added and incubated for 30 min at 37 °C. Plates were washed six more times, followed by the addition of 100 μL of TMB substrate and incubation at room temperature for 20 min. Developed ELISA spots were imaged using the PictImager™ (sciREADER CL2 colorimetric plate reader), and spot intensities quantified using Pictorial™ analysis software.

### 2.4 IFN-*γ* test

IFN-γ test was performed as previously described (11). Briefly, heparinized whole blood samples were collected from cattle, and for each sample, 300 μL of blood containing approximately 600,000 polymorphonuclear cells was dispensed into 96-well round-bottom plates. The samples were stimulated for 18 h at 39 °C in a 5% CO₂ atmosphere with 3 μg per well of either MAP sonicated extract, Pokeweed mitogen (PWM), or left unstimulated as a negative control. After incubation, the plates were centrifuged to pellet cells, and the culture supernatant was harvested and stored at −20 °C. IFN-*γ* levels in the culture supernatant were quantified using the Bovigam TB ELISA kit (ThermoFisher Scientific, Waltham, MA, USA).

### 2.5 Positive and negative agreement

As the IFN-*γ* test served as a non-reference comparator, positive percent agreement (PPA), negative percent agreement (NPA), and overall agreement were calculated to assess concordance between the tests.

**Table:** 

	IFN-γ test			Total
		+	−	
New Test	+	a	b	a + b
	−	c	d	c + d
Total		a + c	b + d	a + b + c + d

PPA was defined as 100% x a / (a + c), NPA as 100% x d / (b + d), and overall agreement as 100% x (a + d) / (a + b + c + d). These metrics were calculated to evaluate the test’s diagnostic performance in the absence of a recognized reference standard.

### 2.6 Data interpretation

The Pictor PictVet™ MAP IgG Multiplex ELISA results are reported both as spot signal intensities and qualitatively as positive or negative. All five markers were used to determine the outcome of the assay through an algorithm, whereby a sample was considered positive if it tested positive for at least two of the five antigens.

### 2.7 Statistical analysis

Agreement between each assay and the reference method was assessed using the exact binomial version of McNemar’s test, which is recommended for small sample sizes and when discordant counts are low. For each pairwise comparison, discordant results were classified as (b) reference positive and test negative, and (c) reference negative and test positive. The exact two-sided *p* value was calculated using the formula:

$$p=2\times {\left(0.5\right)}^{\left(b+c\right)}$$

## 3 Results and discussion

**Figure 2:**
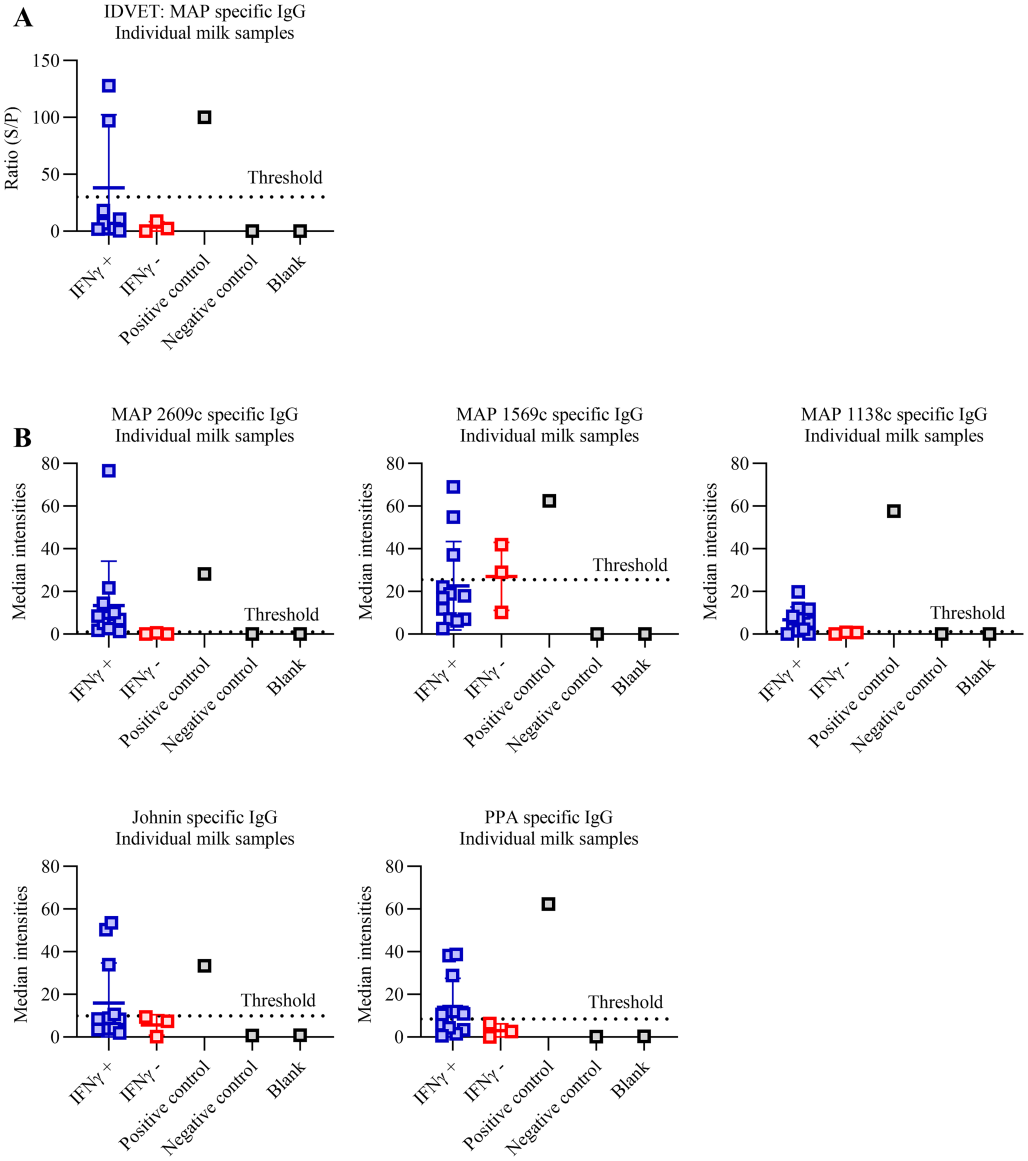
Pictor PictVet™ MAP IgG multiplex ELISA detects MAP-specific IgG antibodies in bovine milk. **(A)** Scatter plot showing MAP-specific IgG responses measured by the IDVET milk ELISA in IFN-*γ*–positive (IFN-*γ*⁺, blue) and IFN-γ–negative (IFN-γ^−^, red) samples, along with positive and negative controls and blanks. The dotted line indicates the assay threshold. **(B)** Median signal intensities for MAP-specific IgG antibodies detected using the Pictor PictVet™ MAP IgG multiplex ELISA in individual milk samples. Antigens tested include MAP2609c, MAP1569c, MAP1138c, Johnin, and PPA. Results are shown for IFN-γ⁺ (blue) and IFN-γ^−^ (red) samples, as well as positive control, negative control, and blank wells. Dotted lines represent the threshold for each antigen based on assay cut-off values. Error bars represent standard deviation (SD).

In this study, well-characterized samples were used, with each sample tested by fecal culture, PCR, antibody ELISA, and, most importantly, IFN-*γ* to accurately identify true negative, suspect, subclinical, and clinical cases of MAP infection (Table 1). The IFN-γ assay was used as a comparator test based on its widely acknowledged role for detecting early, cell-mediated immune responses to MAP infection, often preceding detectable humoral responses (12, 13). To address the limited sensitivity of conventional monoplex ELISAs in identifying subclinical MAP infections (14, 15), the Pictor PictVet™ MAP IgG Multiplex ELISA was developed to detect MAP specific IgG responses using five MAP antigens (MAP2609c, MAP1569c, MAP1138c, PPA, and Johnin) printed in a microarray format (Figure 1A). Antibodies against MAP2609c, MAP1569c, and MAP1138c antigens were previously identified as potential early biomarkers for Johne’s disease (16–18). To account for variability in host immune responses and reduce the likelihood of false positives, a sample was classified as positive if it exceeded the signal threshold for at least two of the five antigens, according to a predefined interpretation algorithm. The multiplex platform clearly distinguished the commercially available MAP-positive reagent from the negative control (Figures 1B,C), with positive samples exhibiting high-intensity signals across multiple antigen spots, and negative samples showing only background reactivity. Using this platform, we evaluated the diagnostic performance of the Pictor PictVet™ MAP IgG Multiplex ELISA in both serum and milk matrices from cattle classified by IFN-*γ* status and compared these results to those from the commercially available IDVET ELISA. As shown in Figure 1D, the IDVET ELISA failed to identify several IFN-*γ*–positive serum samples. In contrast, the Pictor PictVet™ MAP IgG Multiplex ELISA assay demonstrated that specific antigens, particularly MAP2609c, MAP1138c, and PPA, could differentiate between IFN-*γ*–positive and –negative animals (Figure 1E). In the case of Johnin, approximately half of the IFN-γ–positive animals showed responses above the cut-off, while the other half exhibited signal levels comparable to IFN-*γ*–negative samples. Interestingly, MAP1569c produced strong responses in both IFN-γ–positive and –negative samples, indicating high immunogenicity but potentially limited specificity as a MAP biomarker. We extended the evaluation to the milk matrix, where similar patterns were observed (Figure 2). As shown in Figure 2A, the IDVET ELISA failed to detect several IFN-γ–positive samples. In contrast, the Pictor PictVet™ MAP IgG Multiplex ELISA milk assay identified IgG responses against multiple antigens, particularly MAP2609c and MAP1138c (Figure 2B).

**Figure 3:**
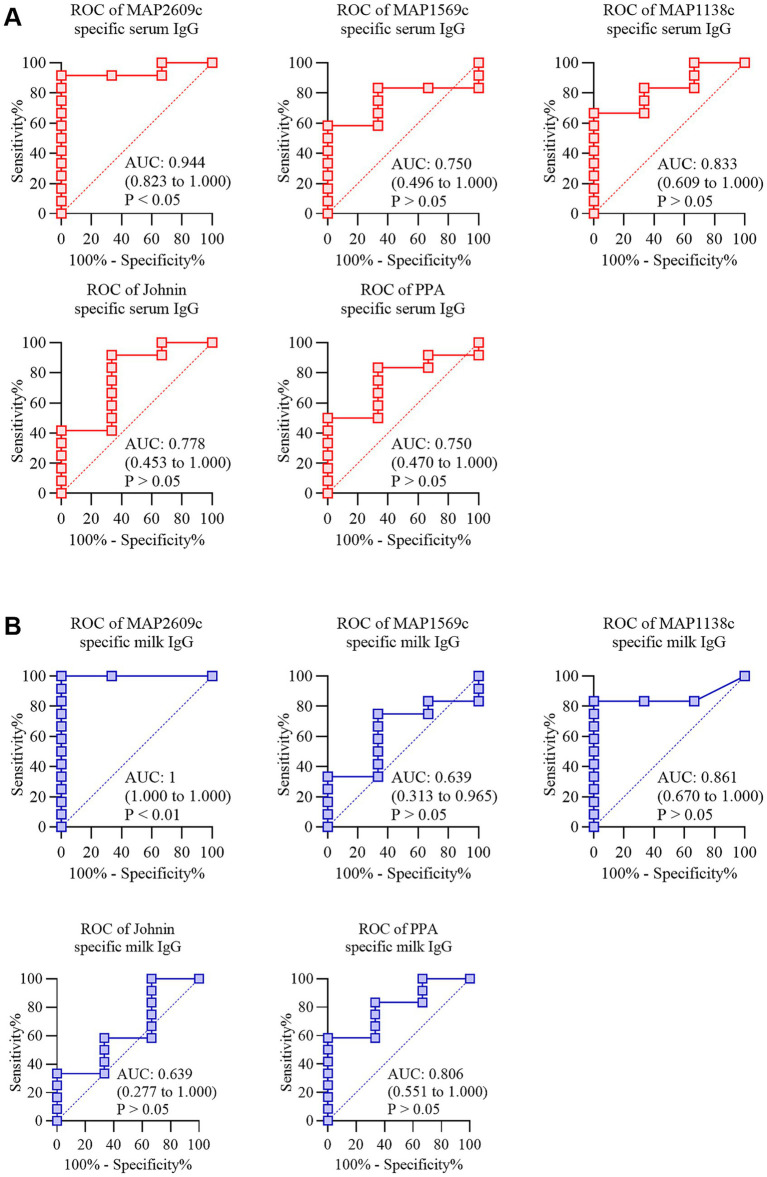
Receiver operating characteristic (ROC) analysis of the Pictor PictVet™ MAP IgG Multiplex ELISA. ROC curves were generated for 15 serum samples **(A)** and 15 milk samples **(B)** collected from 15 cows to assess the diagnostic performance of the Pictor PictVet™ MAP IgG Multiplex ELISA relative to IFN-γ classification. Each curve plots sensitivity versus 100 minus specificity and displays the area under the curve (AUC), 95 percent confidence interval, and corresponding *p*-value, indicating the statistical significance of discrimination from random classification. The dashed diagonal represents the line of no discrimination (AUC = 0.5).

To further examine the discriminatory performance of each antigen, we carried out ROC analysis using the validation set of 15 serum and 15 milk samples (Figure 3). For serum, MAP2609c showed the strongest separation between IFN gamma positive and negative animals with an AUC of 0.944 (95 percent CI 0.823 to 1.000). MAP1138c also performed well with an AUC of 0.833 (95 percent CI 0.609 to 1.000), while Johnin, MAP1569c, and PPA showed moderate discrimination with AUC values of 0.778, 0.750, and 0.750, respectively. In milk, MAP2609c achieved an AUC of 1.000, indicating complete separation of the two groups in this cohort. MAP1138c showed good performance with an AUC of 0.861 (95 percent CI 0.670 to 1.000), followed by PPA with an AUC of 0.806. MAP1569c and Johnin showed lower discrimination, with AUC values of 0.639. The diagonal reference line represents no discrimination. These ROC results align with the predefined two antigen positivity algorithm and support the robustness of the multiplex platform. The ROC analysis shows that several antigens, particularly MAP2609c and MAP1138c, contribute strongly to the assay’s ability to detect MAP specific antibodies in both serum and milk.

The gold-standard methods for confirming MAP infection are fecal culture and PCR (19), both of which were employed in this study to accurately classify infection status. In addition to these methods, we also used IFN-*γ* and IDEXX antibody ELISA testing to classify the cows into true negative, suspect, subclinical, or clinical categories (Table 1). Among the 15 cows in this longitudinal study, 2 were classified as suspect, 7 as subclinical, and 3 as clinical. Notably, IDEXX identified the “suspect” animals as negative, while Pictor PictVet™ identified them as positive, similar to the IFN-*γ* test. Of the 7 subclinical samples, IDEXX identified only 1 as positive and 1 as suspect, misidentifying 5 as negative, whereas Pictor PictVet™ correctly detected 5 out of 7 subclinical samples. Interestingly, IDEXX also failed to detect 1 of the 3 clinical samples. Overall, the IDEXX and IDVET assays demonstrated only 25–42% PPA, both failing to detect several IFN-*γ*–positive animals, highlighting the limitations of conventional monoplex assays in detecting early or subclinical infections. The Pictor PictVet™ MAP IgG Multiplex ELISA serum assay achieved 83% PPA, 100% NPA, and 86.7% overall concordance with IFN-γ classification (Table 2), demonstrating its superior ability to detect early or low-level humoral responses. Performance metrics for milk samples mirrored those of serum, with the Pictor PictVet™ assay maintaining 92% PPA, 100% NPA, and 93.3% concordance with IFN-γ status (Table 3).

**Table 2:** Comparison of MAP antibody detection in serum samples using the Pictor PictVet™ MAP MAP IgG multiplex ELISA, IDEXX ELISA, IDVET ELISA, and IFN-γ assay.

Serum ID#	IFN-γ test	Pictor PictVet™	IDEXX	IDVET
S785	Negative	Negative	Negative	Negative
S486	Negative	Negative	Negative	Negative
S488	Negative	Negative	Negative	Negative
S781	Positive	Positive	Negative	Negative
S1091	Positive	Positive	Negative	Positive
S9514	Positive	Positive	Negative	Negative
S788	Positive	Negative	Positive	Positive
S1044	Positive	Negative	Negative	Negative
S1459	Positive	Positive	Suspect	Negative
S1618	Positive	Positive	Negative	Negative
S1701	Positive	Positive	Negative	Negative
S6607	Positive	Positive	Negative	Positive
S2222	Positive	Positive	Positive	Positive
S6739	Positive	Positive	Positive	Negative
S6878	Positive	Positive	Suspect	Negative
PPA		83	25–42*	33
NPA		100	100	100
% Concordance		87	40.0–53*	47

*The range of PPA and concordance for the IDEXX assay was calculated by treating samples classified as suspect as positive in one analysis and as negative in a separate analysis.

**Table 3:** Comparison of MAP antibody detection in milk samples using the Pictor PictVet™ MAP IgG Multiplex ELISA, IDEXX ELISA, IDVET ELISA, and IFN-γ assay.

Milk ID#	IFN-γ test	Pictor PictVet™	IDEXX	IDVET
M785	Negative	Negative	Negative	Negative
M486	Negative	Negative	Negative	Negative
M488	Negative	Negative	Negative	Negative
M781	Positive	Positive	Negative	Negative
M1091	Positive	Positive	Negative	Negative
M9514	Positive	Positive	Negative	Negative
M788	Positive	Positive	Positive	Positive
M1044	Positive	Negative	Negative	Negative
M1459	Positive	Positive	Negative	Negative
M1618	Positive	Positive	Negative	Negative
M1701	Positive	Positive	Negative	Negative
M6607	Positive	Positive	Negative	Negative
M2222	Positive	Positive	Positive	Positive
M6739	Positive	Positive	Positive	Positive
M6878	Positive	Positive	Suspect	Negative
PPA		92	25–33*	25
NPA		100	100	100
% Concordance		93	40–47*	40

^*^The range of PPA and concordance for the IDEXX assay was calculated by treating samples classified as suspect as positive in one analysis and as negative in a separate analysis.

McNemar’s test was used to determine whether each assay differed from the reference method in how it classified MAP positive and MAP negative samples (Table 4). The test examines the number of discordant results within matched pairs and is suitable for small sample sizes such as in this study, where the sample size is 15 per sample matrix. For each comparison, discordant outcomes were grouped as false negative (reference positive and test negative) or false positive (reference negative and test positive). For serum samples, the Pictor PictVet™ assay did not differ significantly from the reference method (2 false negatives, 0 false positives; exact *p* = 0.50). In contrast, the IDEXX and IDVET assays showed significant differences, with seven and eight false negative results, respectively, and no false positives. For milk samples, the Pictor PictVet™ assay also showed no significant difference from the reference (1 false negative, 0 false positives; *p* = 1.00), while IDEXX and IDVET again showed significant disagreement, with eight and nine false negatives, respectively. These results indicate that the Pictor PictVet™ assay showed closer agreement with the IFN-*γ* classification than either the IDEXX or IDVET ELISAs in both serum and milk. This observation is consistent with the higher PPA values reported above. Overall, the findings reported in this study underscore the advantage of multiplex platforms like Pictor PictVet™ in detecting MAP infections that may otherwise be missed. While the IFN-γ assay may be used for early detection, it does not directly confirm the presence of MAP bacteria. Therefore, the comprehensive approach using fecal culture, PCR, and antibody ELISA in this study ensured accurate classification of infection status, which we believe is important for a robust evaluation of our multiplex ELISA’s performance.

**Table 4:** Exact McNemar test comparing each assay with IFN-γ for serum and milk samples.

Sample type	Comparison against IFN-γ assay	False negative*	False positive**	Exact McNemar *p*-value	Interpretation
Serum	Pictor PictVet™	2	0	0.50	Not significant
	IDEXX	7	0	0.0156	Significant
	IDVET	8	0	0.0078	Significant
Milk	Pictor PictVet™	1	0	1.00	Not significant
	IDEXX	8	0	0.0078	Significant
	IDVET	9	0	0.0039	Significant

*Test negative and IFN-γ assay positive. **Test positive and IFN-γ assay negative.

Notably, the majority of IFN-*γ*–positive milk samples surpassed the two-antigen positivity threshold, highlighting the assay’s robustness across sample types (Table 3). This performance is particularly noteworthy given the lower IgG concentration in milk and the well-documented challenges of milk-based antibody detection (20–22). Our results suggest that the multiplex platform offers reliable detection of MAP-specific antibodies in milk without compromising specificity, further validation in larger and more diverse sample cohorts is warranted.

The strength of the IFN-*γ* assay is its ability for early detection of MAP infection, particularly during the cell-mediated phase that precedes humoral response (12). Consistent with this, several IFN-γ–positive animals were negative by both IDEXX ELISA and IDVET ELISA (Tables 2, 3). The Pictor PictVet™ assay, however, showed better concordance with IFN-γ status in both serum and milk, indicating its enhanced ability to detect early or low-level humoral responses. We hypothesize that the multiplex format, combined with the dual-antigen positivity algorithm, improves the sensitivity during the subclinical phase while minimizing false positives due to non-specific single-antigen reactivity. These characteristics support the advantages of multiplex immunoassays in chronic infections like Johne’s disease, where immune responses evolve dynamically and vary across individuals. Our assay is highly customizable. If new biomarkers are identified in the future, additional MAP targets can be tested and incorporated to further enhance diagnostic performance in detecting early subclinical MAP infections.

Cross-reactivity with non-pathogenic environmental mycobacteria is a common challenge in serological assays for MAP, as it can lead to false positives and reduce the reliability of diagnostic tests (23). To address this issue, we carefully selected recombinant MAP antigens that are highly specific to MAP and have been demonstrated to provoke robust immune responses in MAP-infected cattle, particularly during the early, subclinical phase when antibody levels are typically low. The three recombinant MAP antigens included in our multiplex assay, which are MAP2609c, MAP1569c, and MAP1138c, were identified in previous studies that screened a large set of recombinant MAP proteins for their reactivity in infected cattle (17, 24). These studies used protein microarrays and immunoassays to evaluate the immune response to MAP antigens at various stages of infection. MAP2609c and MAP1569c were found to provoke strong immune responses in early MAP infections, while MAP1138c (P22) was shown to stimulate both humoral and cellular immune responses. These antigens were specifically chosen for their ability to detect MAP infection even during the subclinical phase, when antibody responses are not yet detectable by conventional serological tests. Importantly, these antigens were identified without the use of *M. phlei* absorbent, further confirming their MAP-specific reactivity and minimizing concerns about cross-reactivity. Given the high specificity of these MAP-specific antigens, pre-treatment of serum samples with *M. phlei* or other absorbents to remove cross-reactive antibodies is not necessary. This reduces the need for additional sample treatment, streamlining the assay while maintaining its accuracy and efficiency.

To further ensure the specificity of the assay, several additional steps were implemented. Each biomarker was set with a high cut-off to achieve 100% specificity, and we applied an algorithm that requires a sample to test positive for at least two of the five biomarkers in order to be classified as positive. Often, setting the cutoff high can improve specificity, but it may compromise sensitivity, which we observed with each individual biomarker. However, by multiplexing 5 antigens, sensitivity improves significantly. For instance, if MAP2609c misses a MAP infection, the other biomarkers, can still detect MAP antibodies, ensuring a more reliable detection. This dual-antigen positivity requirement, combined with a high cut-off for each biomarker, helps to reduce the risk of false positives, ensuring that only samples with strong reactivity to multiple MAP antigens are classified as positive. A notable advantage of the Pictor PictVet™ MAP IgG Multiplex ELISA is its cost-effectiveness, particularly in terms of equipment requirements. Unlike many other multiplex assays that necessitate specialized instruments, our assay can be easily integrated into existing diagnostic laboratories, as it requires only a PictImager™ plate reader, which is comparable in price to those used for traditional ELISA assays. The assay workflow is similar to conventional ELISA, requiring only a plate washer for operation, making it familiar to labs already performing these tests. This streamlined approach minimizes the need for additional investment, making the Pictor PictVet™ MAP IgG Multiplex ELISA a practical option for large-scale screening. In addition to being cost-effective, the assay offers enhanced sensitivity and specificity through multiplexing, especially for early detection of MAP infection, which can lead to better herd management and long-term savings in disease control. As the assay becomes more widely adopted, we expect that economies of scale will further reduce the cost of reagents and equipment, improving its feasibility for broader use in both research and commercial applications.

It is important to acknowledge that this study was conducted on a limited sample size (n = 30, with 15 cows tested in both serum and milk), which may limit the broader applicability of the results. To further validate the diagnostic performance of the Pictor PictVet™ MAP IgG Multiplex ELISA and refine antigen selection, future studies should include larger and more diverse cattle populations, along with longitudinal monitoring of infection status. These studies will be crucial for confirming the assay’s performance across various stages of MAP infection. In addition, we are planning a larger external validation study that will involve multiple farms across New Zealand. This validation study will provide a more robust sample set, which will strengthen the overall findings and help to further optimize antigen selection.

## 4 Conclusion

The Pictor PictVet™ MAP IgG Multiplex ELISA demonstrated superior performance compared to the conventional monoplex ELISA in both serum and milk matrices, achieving higher PPA while maintaining 100% NPA in this study. By targeting multiple MAP antigen-specific antibodies and employing an algorithm-based interpretation, the assay enables robust detection of early-stage and subclinical infections. The assay’s compatibility with milk samples also offers a practical, non-invasive solution for herd-level surveillance. While these results suggest that the multiplex approach may offer benefits over conventional ELISAs, the findings are based on a limited dataset and should be interpreted with caution. Validation in larger and independent cohorts will be required to confirm the diagnostic performance and utility of the assay across different stages of MAP infection.

## Data Availability

The original contributions presented in the study are included in the article/supplementary material, further inquiries can be directed to the corresponding author.
